# Experimental approach to the dislodging effect and the mortality of a pesticide in the yellow scorpion *Tityus serrulatus*

**DOI:** 10.1371/journal.pone.0289104

**Published:** 2023-07-27

**Authors:** Gabriel Pimenta Murayama, Bruna Barbosa, Rodrigo Hirata Willemart

**Affiliations:** 1 Laboratório de Ecologia Sensorial e Comportamento de Artrópodes (LESCA), Escola e Artes Ciências e Humanidades, Universidade de São Paulo, São Paulo, Brazil; 2 Programa de Pós-Graduação em Zoologia, Instituto de Biociências, Universidade de São Saulo, São Paulo, Brazil; Instituto Butantan, BRAZIL

## Abstract

Accidents with scorpions are a problem in several regions of the world. In Brazil, the number of accidents is sometimes higher than 160k/year, and the responsible for most accidents and deaths is the yellow scorpion *Tityus serrulatus*. Unfortunately, there are few publications testing the effectiveness of most of the products for chemical control of scorpions. Using the pesticide Bifentol, we tested: I–the effect of the pesticide on the mortality of *T*. *serrulatus*, II–whether the scorpion avoids areas with pesticide and, III–whether it leaves the shelter if pesticide is applied. In the first experiment, we applied pesticide or water on the dorsal region of the scorpion or substrate according to treatment. For five days we noted whether the scorpion slide (dead) or clung to the substrate (alive) after turning the arena vertically to left and right. After five days, no pesticide-treated scorpions were alive while all water-treated scorpions were. In the second experiment, we placed two shelters, applied pesticide and/or water inside the shelter. We then released a scorpion on the opposite side. We scored latency to enter one of the shelters and the choice made by the scorpions. We did not find differences in latency or in the choice made. In the third experiment, we applied the pesticide or water to the shelter where the scorpion was being maintained, and, on the following day, we recorded whether the scorpion had left the shelter. None of the scorpions left the shelters and only one died. Thus, we obtained evidence that a pesticide can kill scorpions, but we did not find a dislodging effect.

## Introduction

Scorpionism is a problem in several parts of the world [1], Brazil being one of the countries with more accidents and deaths by scorpions. In the last 20 years the number of reported accidents has increased, sometimes reaching more than 169k/year [2] and hundreds of deaths in this decade. Most of the accidents and deaths in Brazil are caused by three species in the family Buthidae: *Tityus stigmurus*, *Tityus bahiensis* and *Tityus serrulatus*, which are commonly found in urban environments.

Different approaches have been used to control scorpions in Brazil, the most common being manual collection indoors and outdoors, a method typically used by health agents. There is also evidence that some predators have potential for biological control: Jared et al. [3] have shown that the toad *Rhinella icterica* is resistant to the venom of *T*. *serrulatus* and that it may feed on the scorpion. The domestic hen *Gallus gallus domesticus* does not stop feeding on individuals of *T*. *serrulatus* despite being stung and does not die from stings [4]. An additional method may be the use of pesticides.

Spraying pesticides against scorpions, although common, has not often had its efficiency tested. Two field studies on the subject had data collected by house holders: Ramsey et al. [5] assessed scorpion prevalence by regular reports, before and after spraying houses. The authors have suggested that four tested formulations of pyrethroid (bifenthrin, cyfluthrin, deltamethrin and K-Othrine) reduce the number of scorpions observed. Albuquerque et al. [6], with a similar method, have applied the pesticide Demand 2.5 and have suggested it is not efficient. In both these pioneer and important papers, one cannot tell if scorpions not seen moved away or died following the application of the pesticide. Santos & Albuquerque [7] have shown, in laboratory experiments, that the pesticide Bifentol (bifenthrin 20%) did not kill the scorpion *T*. *stigmurus*. Only immature individuals avoided areas with pesticides and, although scorpions have had behavioral reactions to the pesticide, they would regain their normal posture after a few days. Brito-Almeida et al. [8] have evaluated how time elapsed after application of the pesticide Fipronil affects mortality rates in *T*. *stigmurus*, finding that within three hours mortality is high and that after 24h it is ineffective.

Although it has been speculated, it is unknown if scorpions will avoid entering shelters previously sprayed with pesticides. It is also unknown if they will leave their shelters once they are sprayed with pesticides. Such variables are especially important since they can be very relevant in deciding whether or not to use pesticides, because scorpions that avoid shelters with pesticides will likely wander more and, therefore, increase the chances of contact with humans. Another important but often overlooked factor is a consequence of pesticides being applied during the day when scorpions are usually sheltered. Pesticides are sprayed in putative scorpion shelters, so that the pesticide can either contact the scorpion’s body directly, or only its surroundings. It is unexpected that scorpions will react the same way in these different scenarios. But this is also unknown. Furthermore, there has been no systematic and quantitative information for controlling the yellow scorpion *T*. *serrulatus*, a parthenogenic species responsible for most accidents in Brazil [9, 10]. Despite accidents with this species being long known [11, 12], very few studies on strategies to minimize encounters have been published. Specifically, there is not a single paper testing a pesticide against it.

Therefore, here we tested: I–the effect of pesticide (on the body or the substrate) on the posture and mortality of *T*. *serrulatus*, II–if the scorpion avoids entering areas with pesticide and III–if the scorpion leaves its shelter if pesticide is applied.

## Material and methods

### General procedures

For the first experiment, we used *T*. *serrulatus* collected in the cities of Santa Gertrudes (22°27’10.4"S 47°31’46.7"W) and Botucatu (22°53’50.2"S 48°26’44.0"W), in the State of São Paulo, Brazil. For the second experiment, we collected individuals of *T*. *serrulatus* (distinct from the ones used in Experiment 1) in Botucatu (22°53’50.2"S 48°26’44.0"W) and Tupã (22°55’22.0"S 47°02’54.6"W). Both cities are in São Paulo State, Brazil. We only used adult scorpions (~7 cm). Before the experiments, we maintained scorpions in groups in containers (20 cm diameter, 8cm height), with water available *ad libitum* in wet cotton balls and cardboard shelters. The scorpions were in a dim room under a natural light regime. We randomly assigned individuals to treatments.

For all the experiments, we used the pesticide Bifentol 200 SC (pyrethroid with bifenthrin as an active ingredient), which is indicated to kill scorpions. We chose Bifentol because it is easily available and because it had been used in a previous study (7). We followed the recommendations of the company and prepared a solution of 4mL of Bifentol / L of distilled water. We have conducted all the preparation procedures and the experiments in a fume hood. To simulate the period when 103 the pesticide is applied by company workers, we ran the trials between 8 and 18h. Temperature during the experiments was ~23°C. We used the arenas only with a single scorpion and all material used was discarded after each trial.

#### 1. Effect of pesticide: Site of application, posture, and mortality

To test if scorpions would die or not under the effects of the pesticide, we used individual arenas (8 cm diameter and 5 cm height) for each scorpion. Each arena had a layer of shrink wrap on the bottom and walls, so that the pesticide would not contact the arena directly. We then placed a layer of filter paper on the bottom of the arena. To minimize the concentration of pesticide in the arena we made 5 small holes on the lid to improve the ventilation. Additionally, we attached a cotton ball to the cover to provide humidity without wetting the filter paper.

To apply the pesticide, we used two spray bottles, for the following treatments: pesticide on the body (n = 12 individuals), distilled water on the body (n = 11), pesticide on the substrate (n = 12), distilled water on the substrate (n = 12), totalizing 47 scorpions. We discarded the first spraying to make sure that the spray mechanism was working well. We removed the cover of the arena, and sprayed 0,0689g (mean; SD = 0.0089; n = 10, we weighed a posteriori), at a distance of 5 cm, either onto the filter paper or onto the mesosoma of the scorpion depending on the treatment. For substrate treatments, we gently removed the scorpion before the application. Spraying was done by the same person in every trial, to avoid intra-individual differences.

**Fig 1:**
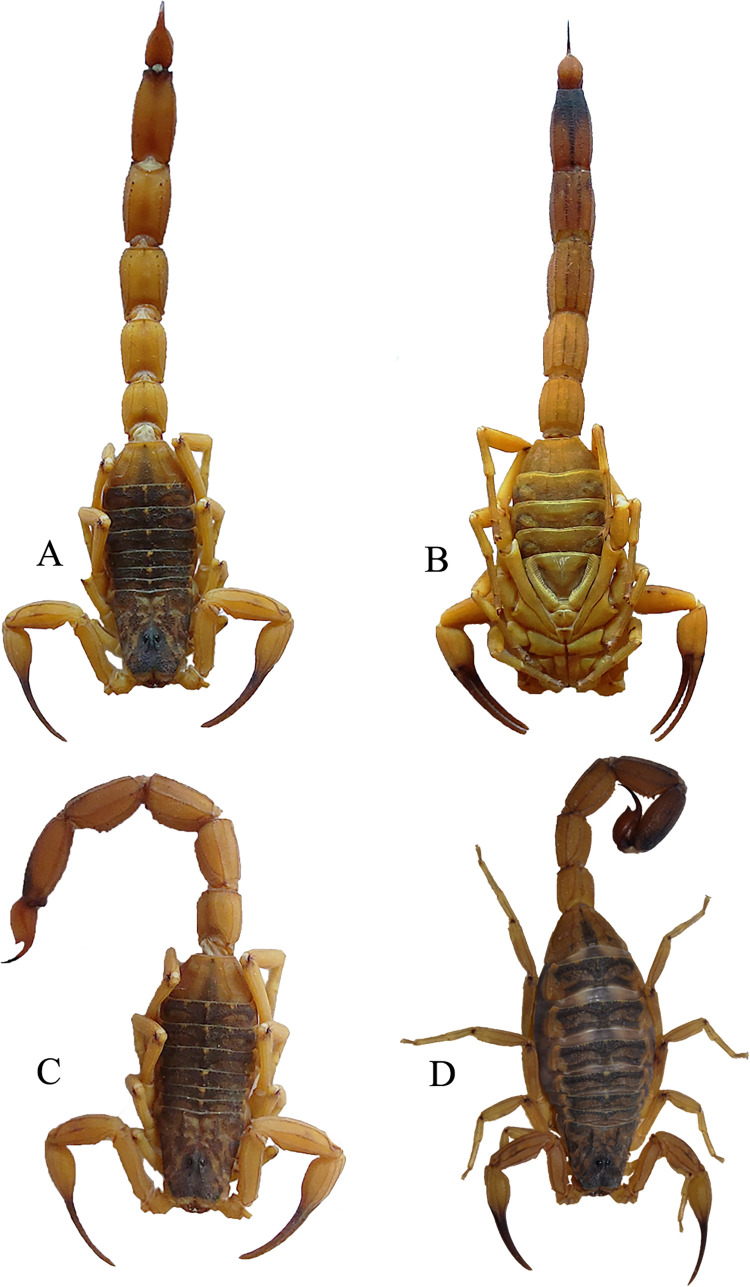
Postures of the *Tityus serrulatus* in the first experiment of the effect of pesticide. A. laying on the ground, B. in *dorsal decubitus*, C. stretched out with a flexed metasoma and, D. normal. The animals in the pictures were not the ones used in the experiment.

We monitored the scorpions for a period of 5 days. In the first day, after application, we observed scorpion behaviors for the first 15 min and then registered animals´ posture after 30 min, 1h, 1h30, and 2h from time zero. Then, for 4 days, we observed the animals once a day at ~16h. We registered whether the scorpion was: A. laying on the ground, B. in *dorsal decubitus*, C. stretched out with a flexed metasoma and, D. normal. (Fig 1). These categories were a proxy of scorpion state during the period of the experiment, in which Fig 1D correspond to live animal whereas Fig 1A–1C correspond to dead or dying animals. We would then move the arena from the horizontal to a vertical position up to 6 times and noted whether the scorpion slid (dead) or held itself to the substrate (alive). This was to confirm if an animal was dead. When we finished monitoring all 136 the individuals, we watered all the cotton balls (1.5mL) through a hole on the cover of the arena, with the help of a dropper.

#### 2. Choice experiment: test of pesticide avoidance

Here we have tested whether a scorpion outside a shelter would avoid entering a pesticide impregnated shelter. To do that, we first individualized the scorpions in a dark room. For the tests, we used an arena (15 cm height and 25 cm diameter) where we placed two shelters ~5 cm apart, each with a wet cotton ball on an aluminum case to prevent water to leak. Both shelters were in contact with the wall of the arena. We also introduced, on the bottom of the shelter, a plastic sheet with a 1 x 4 cm filter paper strip on top, where we would apply either pesticide or distilled water (control). The plastic sheet was to prevent pesticide or water to leak on the arena. In each test, we introduced a scorpion in a vial and let him acclimate for 3 min equidistantly from the shelters. During this period, we used two pipettes to apply a droplet of distilled water or pesticide on the filter paper strip previously mentioned (pesticide droplet = 0,0350 g; SD = 0.002; n = 6; measured a posteriori). We then removed the vial and left the scorpion for 30 min in the arena, scoring latency to enter one of the shelters and the choice made. Once the scorpion was in the shelter, we waited 2 min before scoring a choice. We randomized the position of the shelters with control/treatment and the position of the arenas. We used 30 individuals from Botucatu (distinct from the ones in Experiment 1) and 40 from Tupã. Fume hood and its lights were left on to maximize the chances that scorpions would look for shelter.

#### 3. Dislodging experiment

Here we have tested whether sheltered scorpions will move away if the pesticide is applied to the shelters. We used a circular arena (20 cm diameter, 8 cm height), with squared paper on the bottom to allow us to control for distance between the scorpion and the shelter. The arena was as in experiment 2 but with a single shelter in contact with the arena wall and an additional cotton ball outside the shelter, in the extreme opposite to the shelter. The filter paper strip where water or pesticide were applied were as in experiment 2.

We introduced the scorpion in the opposite region (in relation to the shelter) of the arena, covered it with a vial, let the scorpion acclimate for 5 min, and started scoring. Twenty scorpions were tested with pesticide and another 20 with distilled water (same procedure as experiment 2), each individual being monitored for two days. On the first day, we applied the pesticide or water and observed the animals for 25 min. On the second day, we would register the distance between the animals and the shelter. Because here we wanted to create a friendly environment outside the shelter to maximize the chances of the scorpions getting out of it, we turned off the fume hood and its lights.

### Statistical analyses

We used the software BioEstat 5.0 and tested for normality using a Shapiro-Wilk test. In the first experiment we used a chi-square to compare the mortality between treatments. In the second experiment, we used (1) a chi-square test to compare the number of choices between the two shelters (with and without pesticide) and (2) a Mann-Whitney test to compare latency to choose between the shelters.

## Results

### Effect of pesticide: Site of application, posture and mortality

During the first 15 min, we observed the following behaviors only in individuals treated with pesticide: clean the pedipalps (n = 7: 4 substrate and 3 body), metasoma spasms (n = 9: 1 substrate and 8 body), walk fast (n = 9: 2 substrate and 7 body), attempt to climb the wall (n = 5: 2 substrate and 3 body), body trembling (n = 2: 1 substrate and 1 body) and mesosoma off the ground (n = 4: all in the substrate).

After ~15 min, all the scorpions in the water treatments remained in their usual posture (= “hanging on the cotton of the lid” + “normal"). After 30 min, we found two individuals in the treatment pesticide on the body in *dorsal decubitus*. After 2 h, in the pesticide treatments, 10 individuals were found lying on the ground (Fig 1A) and three individuals were in *dorsal decubitus* (Fig 1B).

**Fig 2:**
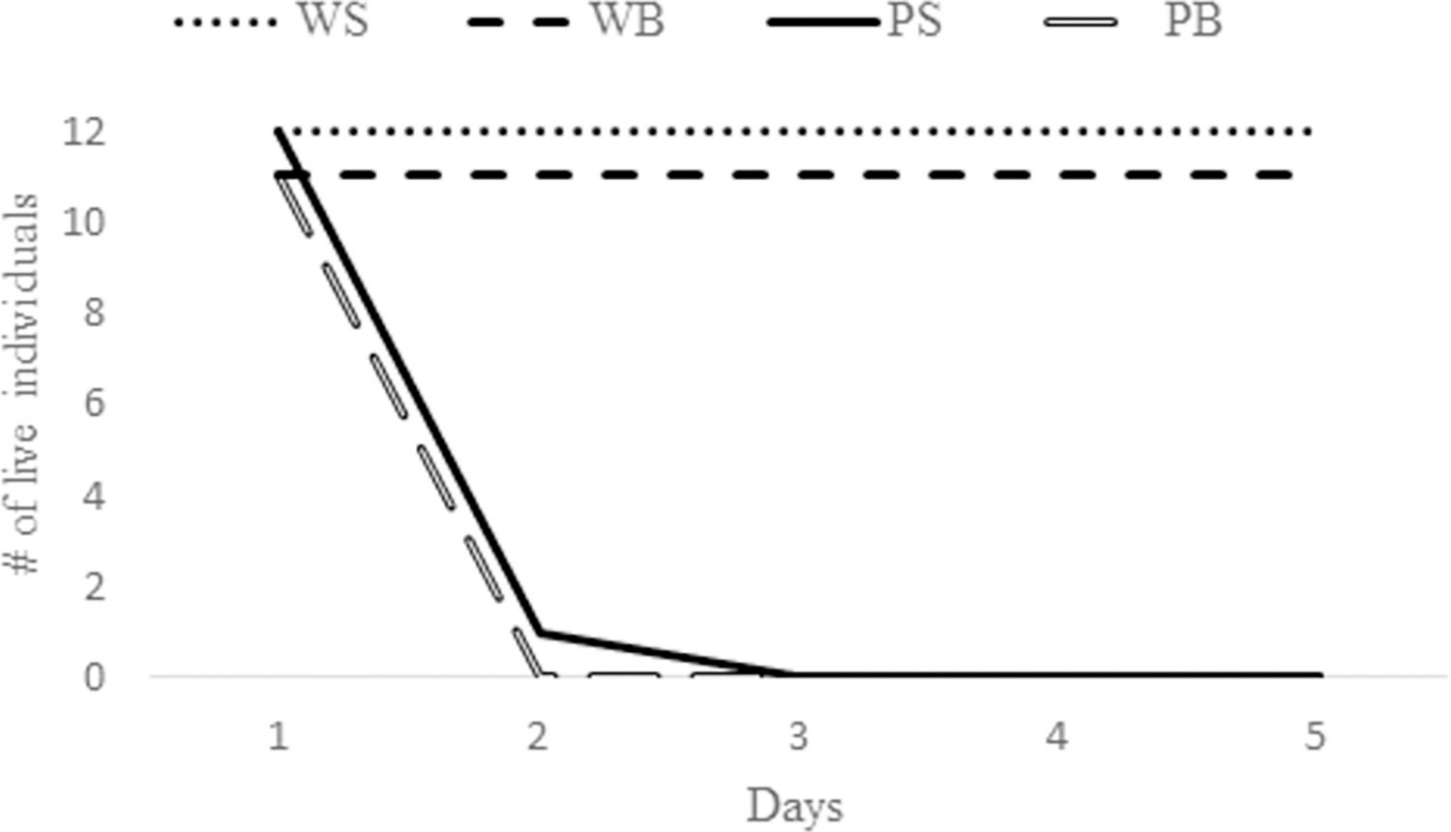
Number of responsive *Tityus serrulatus* following application of pesticide or water. WS = water on the substrate; WB = water on the body; PS = pesticide on the substrate; PB = pesticide on the body.

Finally, after five days, all scorpions treated with water were alive, whereas all the scorpions treated with pesticide were dead (Fig 2; S1 Table).

### Choice experiment: Test of pesticide avoidance

We have observed four individuals stop moving abruptly at the entrance of the pesticide shelter and choosing the control shelter. One individual did the opposite, choosing the treatment shelter. After choosing one of the shelters, all the individuals remained in it until the end of the trial. Thirty-one individuals have chosen the treatment shelter and 39 the control one (x2 = 0.46; df = 1; p = 0.61). We also found no difference in the latency to choose either of the shelters (median difference in time spent to choose water treatment = 652 s, min = 19 s, max = 1656 s; median difference in time spent to choose pesticide treatment = 653 s, min = 22 s, max = 1620 s, Mann-Whitney test: U = 532; p = 0.39; S2 Table).

### Dislodging experiment

None of the scorpions left the shelters (n = 20) and only one individual died, in the pesticide treatment.

## Discussion

Our data suggest that, in a small and covered environment, the pesticide Bifentol can be efficient in killing the scorpion *T*. *serrulatus*, both if applied on the body and the substrate. However, we did not find evidence that scorpions avoid entering a shelter with pesticide at least when there is light outside the shelter. Finally, we also found no dislodging effect in the conditions of our experiment.

The pesticide tested may kill scorpions, a result that is contrasting with two previous studies with other scorpion species and pesticides [6, 7]. Because in these previous works the pesticides also had bifenthrin among their ingredients, the differences found may have resulted from how pesticides were applied and/or distinct resistance between *T*. *stigmurus* and *T*. *serrulatus* to them. It may also be that bifenthrin only kills if scorpions are in covered and confined areas [7 do not mention covers in the arenas they have used]. Besides, although bifenthrin has a low volatility as other pyrethroids [13, 14], in a covered environment it would maximize the intoxication. We did not test the effect of the pesticide in open areas and the environmental context may be determinant. Recently, Brito-Almeida et al. [8] have shown that the scorpion *T*. *stigmurus* died when in contact with areas treated with the pesticide Fipronil. They also showed that mortality rate decreased after the scorpion had been exposed to areas with Fipronil for eight hours. The method and the pesticide were different from the one used here and in a previous study with *T*. *stigmurus* [Bifentol– 7]. Maybe Fipronil is stronger than Bifentol since continuous exposure was not necessary to kill the scorpion *T*. *stigmurus*.

We have shown sublethal effects–neurotoxic action that may cause muscle atony among other symptoms [15, 16] of the pesticide in *T*. *serrulatus*. Spasms and trembling have also been observed in *T*. *stigmurus* [7]. In our study [but not in 7], all animals showing these behaviors eventually died, so these behaviors may be used as proxies of the killing effect of the pesticide at least in conditions similar to our experiment.

We expected the treatment ‘pesticide on the body’ to cause faster changes in posture and higher mortality than ‘pesticide on the substrate’, but we found no differences between these treatments. This might have been because scorpions have several body parts where chemicals can penetrate the body in the ventral region: mouth, opening of book lungs, chemoreceptors on the tarsi and pecten [17–19].

Crossing the results of the three experiments, it may be that pesticides may only repel and kill scorpions when in contact with them. In experiment I, we applied the pesticide either on the body or on the filter paper where the scorpion had all its tarsi on. We noticed, in the first 15 min, that they were trying to avoid the pesticide, moving away from it by walking fast and attempting to climb the wall. They could not get out of the arena (because it was covered) and eventually all died. In experiments II and III, we applied pesticide to a thin strip of filter paper in the shelter. The scorpion did not have to step on it. In both these experiments, the scorpions ignored the pesticide, which did not kill or cause any observable differences in posture or behavior. These results have implications for the use of pesticides in scorpion control and may explain some reports that pesticides are not efficient. If this explanation is right, it means that, in the field, pesticides sprayed into holes and creeks may have no effect because they often do not contact the scorpion. Maybe volatiles per se do not harm these animals. An alternative and non-exclusive hypothesis is that the quantity of pesticide mattered in our trials, since in experiments II and III we used more pesticide than in experiment I.

There are examples of arachnids avoiding areas with toxic/repellent chemicals [spider: 20, scorpion: 21]. However, we did not find evidence that the scorpion avoids the pesticide in the conditions of our experiment. Two alternative/complementary hypotheses have to be mentioned. The first one is that scorpions did not detect the chemicals in experiments II and III, which would explain why they ignored the pesticide. But scorpions have several chemoreceptors (pectens, chemosensory hairs and probably the constellation array) and can detect both chemicals on the substrate [22–24] or volatiles [25, 26]. It is therefore unlikely that they have not detected the pesticide. We cannot discard the possibility of a lower concentration of pesticide in the air in experiments II and III, because the arena was larger. But still, the pesticide was possibly concentrated in the shelter. The second hypothesis is that scorpions in experiment II may also have faced a trade-off between remaining exposed, under light and away from the pesticide or sheltered, in the dark, but close to the pesticide Scorpions are nocturnal animals and sensitive to light [27], preferring shaded to bright areas [28–30]. However, in experiment III, when there was no light outside the shelter, scorpions also remained in it. Based on the evidences provided above, we do not find support for the ideas that scorpions did not detect pesticides and that scorpions were avoiding light. We did find evidence, however, that pesticide volatiles may not be a main issue depending on the quantity and concentration.

It is a common saying that pesticides may have a dislodging effect on scorpions, but this was not supported by our data of experiments 2 and 3, when the scorpions could avoid contact with the pesticide. Because we had a plastic sheet underneath the filter paper with the pesticide, its concentration was probably higher than it would be if a similar quantity was applied in a natural situation where pesticide could percolate through the soil, but the scorpions were not repelled. Although this sounds like good news for the use of pesticides, the bad news is that pesticide volatiles may not harm the scorpions. Although we have demonstrated that the tested pesticide can kill the scorpions in closed environments when scorpions were in contact with the pesticide, one cannot guarantee that scorpions will be sprayed upon when the pesticide is applied in houses.

Application of pesticide is done during the day, when scorpions are resting. That increases the chances that the animals will not touch the pesticide by walking on it. At night, although they may wander, most will sit and wait for prey. This, foraging behavior also reduces the chances that pesticide will come into contact with the scorpion. Moreover, Brito-Almeida et al. [8] have shown that, at least with *T*. *stigmurus* and the pesticide Fipronil, there is no lethal effect after 24h of application. These factors all reduce the chances of efficiency of a pesticide despite its lethal effect under specific circumstances. More experimental works are needed so that scorpion control can be based on science instead of anecdotical observations.

## Supporting information

S1 Table Mortality of scorpions Tityus serrulatus treated with the pesticide Bifentol or water after five days. “Slided” and “clung” are operational variables for “dead” and “alive”, respectively. PS = pesticide on the substrate; PB = pesticide on the body; WS = water on the substrate; WB = water on the body. The first column correspond to the individual identification of the animals.
(PDF)

S2 Table Latency to choose between shelters with pesticide or water offered simultaneously, in the scorpion Tityus serrulatus. All the values are provided in seconds. The first column correspond to the individual identification of the animals.
(PDF)
