# Supplementary material for: Experimental approach to the dislodging effect and the mortality of a pesticide in the yellow scorpion Tityus serrulatus
Source: PLoS One. 2023 Jul 27;18(7):e0289104. doi: 10.1371/journal.pone.0289104 (PMC10374035; doi:10.1371/journal.pone.0289104)
Supplement: S1 Table — “Slided” and “clung” are operational variables for “dead” and “alive”, respectively. PS = pesticide on the substrate; PB = pesticide on the body; WS = water on the substrate; WB = water on the body. The first column correspond to the individual identification of the animals. (PDF) [file pone.0289104.s001.pdf]

S1 Table.

Day 1

ID	Slided	Clung
PB1		x
WB1		x
PS1		x
WS1		x
PB2	x	
WB2		x
PS2		x
WS2		x
PB3	x	
WB3		x
PS3		x
WS3		x
PB4	x	
WB4		x
PS4		x
WS4		x
PB5	x	
WB5		x
PS5		x
WS5		x
PB6	x	
WB6		x
PS6		x
WS6		x
PB7	x	
WB7		x
PS7		x
WS6		x
PB8		x
WB8		x
PS8	x	
WS8		x
PB9	x	
WB9		x
PS9		x
WS9		x
PB10	x	
WB10		x
PS10	x	
WS10		x
PB11	x	
WB11		x
PS11		x
WS11		x
PB12	x	

PS12		x
WS12		x

Day 2

ID	Slided	Clung
PB1	X	
WB1		X
PS1	X	
WS1		X
PB2	X	
WB2		X
PS2	X	
WS2		X
PB3	X	
WB3		X
PS3	X	
WS3		X
PB4	X	
WB4		X
PS4	X	
WS4		X
PB5	X	
WB5		X
PS5	X	
WS5		X
PB6	X	
WB6		X
PS6	X	
WS6		X
PB7	X	
WB7		X
PS7		X
WS6		X
PB8	X	
WB8		X
PS8	X	
WS8		X
PB9	X	
WB9		X
PS9	X	
WS9		X
PB10	X	
WB10		X
PS10	X	
WS10		X
PB11	X	
WB11		X
PS11	X	

WS11		X
PB12	X	
PS12	X	
WS12		X

Day 3

ID	Slided	Clung
PB1	X	
WB1		X
PS1	X	
WS1		X
PB2	X	
WB2		X
PS2	X	
WS2		X
PB3	X	
WB3		X
PS3	X	
WS3		X
PB4	X	
WB4		X
PS4	X	
WS4		X
PB5	X	
WB5		X
PS5	X	
WS5		X
PB6	X	
WB6		X
PS6	X	
WS6		X
PB7	X	
WB7		X
PS7	X	
WS6		X
PB8	X	
WB8		X
PS8	X	
WS8		X
PB9	X	
WB9		X
PS9	X	
WS9		X
PB10	X	
WB10		X
PS10	X	
WS10		X
PB11	X	

WB11		X
PS11	X	
WS11		X
PB12	X	
PS12	X	
WS12		X

Day 4

ID	Slided	Clung
PB1	X	
WB1		X
PS1	X	
WS1		X
PB2	X	
WB2		X
PS2	X	
WS2		X
PB3	X	
WB3		X
PS3	X	
WS3		X
PB4	X	
WB4		X
PS4	X	
WS4		X
PB5	X	
WB5		X
PS5	X	
WS5		X
PB6	X	
WB6		X
PS6	X	
WS6		X
PB7	X	
WB7		X
PS7	X	
WS6		X
PB8	X	
WB8		X
PS8	X	
WS8		X
PB9	X	
WB9		X
PS9	X	
WS9		X
PB10	X	
WB10		X
PS10	X	

WS10		X
PB11	X	
WB11		X
PS11	X	
WS11		X
PB12	X	
PS12		X
WS12		X

Day 5

ID	Slided	Clung
PB1	X	
WB1		X
PS1	X	
WS1		X
PB2	X	
WB2		X
PS2	X	
WS2		X
PB3	X	
WB3		X
PS3	X	
WS3		X
PB4	X	
WB4		X
PS4	X	
WS4		X
PB5	X	
WB5		X
PS5	X	
WS5		X
PB6	X	
WB6		X
PS6	X	
WS6		X
PB7	X	
WB7		X
PS7	X	
WS6		X
PB8	X	
WB8		X
PS8	X	
WS8		X
PB9	X	
WB9		X
PS9	X	
WS9		X
PB10	X	

WB10		X
PS10	X	
WS10		X
PB11	X	
WB11		X
PS11	X	
WS11		X
PB12	X	
PS12	X	
WS12		X
