# Supplementary material for: Experimental approach to the dislodging effect and the mortality of a pesticide in the yellow scorpion Tityus serrulatus
Source: PLoS One. 2023 Jul 27;18(7):e0289104. doi: 10.1371/journal.pone.0289104 (PMC10374035; doi:10.1371/journal.pone.0289104)
Supplement: S2 Table — All the values are provided in seconds. The first column correspond to the individual identification of the animals. (PDF) [file pone.0289104.s002.pdf]

S2 Table.

	Water treatment
E7	259
E10	1443
E12	72
E17	1361
E20	302
E1	574
E3	382
E5	740
E6	734
E8	927
E9	1141
E13	1196
E21	275
E22	811
E23	652
E28	1457
E30	318
E31	1225
E32	769
E36	1550
E37	1656
E39	571
E42	32
E43	1335
E34	310
E40	1438
E46	1306
E49	19
E52	1402
E53	550
E54	888
E56	460
E58	530
E59	641
E60	425
E61	615
E66	406
E67	491
E69	1304

	Pesticide treatment
E11	837
E2	293
E4	22
E14	1106
E15	839
E16	289
E18	664
E19	433
E24	1358
E25	747
E26	430
E27	270
E29	77
E35	1430
E38	43
E41	505
E44	1280
E45	964
E47	1173
E33	653
E48	1620
E50	802
E51	332
E55	243
E57	620
E62	899
E63	1346
E64	89
E65	1068
E68	432
E70	303
